# Semi-recumbent body position fails to prevent healthcare-associated pneumonia in Vietnamese patients with severe tetanus

**DOI:** 10.1016/j.trstmh.2011.10.010

**Published:** 2012-02

**Authors:** Huynh Thi Loan, Janet Parry, Nguyen Thi Ngoc Nga, Lam Minh Yen, Nguyen Thien Binh, Tran Thi Diem Thuy, Nguyen Minh Duong, James I. Campbell, Louise Thwaites, Jeremy J. Farrar, Christopher M. Parry

**Affiliations:** a Hospital for Tropical Diseases, 190 Ben Ham Tu, District 5, Ho Chi Minh City, Vietnam; b The Wellcome Trust Major Overseas Programme, Oxford University Clinical Research Unit, 190 Ben Ham Tu, District 5, Ho Chi Minh City Vietnam; c Centre for Tropical Medicine, Nuffield Department of Medicine, University of Oxford, John Radcliffe Hospital, Oxford OX3 9DU, UK

**Keywords:** Tetanus, Healthcare-associated pneumonia, Body position, Semi-recumbent, Tracheostomy, Vietnam

## Abstract

Healthcare-associated pneumonia (HCAP) is a common complication in patients with severe tetanus. Nursing tetanus patients in a semi-recumbent body position could reduce the incidence of HCAP. In a randomised controlled trial we compared the occurrence of HCAP in patients with severe tetanus nursed in a semi-recumbent (30°) or supine position. A total of 229 adults and children (aged ≥1 year) with severe tetanus admitted to hospital in Vietnam, were randomly assigned to a supine (n = 112) or semi-recumbent (n = 117) position. For patients maintaining their assigned positions and in hospital for > 48 h there was no significant difference between the two groups in the frequency of clinically suspected pneumonia [22/106 (20.8%) vs 26/104 (25.0%); p = 0.464], pneumonia rate/1000 intensive care unit days (13.9 vs 14.6; p = 0.48) and pneumonia rate/1000 ventilated days (39.2 vs 38.1; p = 0.72). Mortality in the supine patients was 11/112 (9.8%) compared with 17/117 (14.5%) in the semi-recumbent patients (p = 0.277). The overall complication rate [57/112 (50.9%) vs 76/117 (65.0%); p = 0.03] and need for tracheostomy [51/112 (45.5%) vs 69/117 (58.9%); p = 0.04) was greater in semi-recumbent patients. Semi-recumbent body positioning did not prevent the occurrence of HCAP in severe tetanus patients. [Clinical Trials.gov Identifier: NCT01331252]

## 1 Introduction

Tetanus is an important cause of morbidity and mortality throughout the developing world. Despite the availability of an effective vaccine, an estimated one million cases of tetanus still occur each year.^1^ The principal causes of death in tetanus are respiratory failure and cardiovascular dysfunction secondary to autonomic instability.^2^ The ability to be able to perform a tracheostomy and mechanically ventilate patients has contributed to a significant reduction in mortality due to respiratory failure^3^, ^4^, ^5^ but leads to an increase in the frequency of healthcare-associated pneumonia (HCAP).^6^, ^7^ The management of patients with pneumonia is complicated by diagnostic difficulties and the development of resistance to commonly used antimicrobial agents, problems particularly acute in resource-limited settings. Simple and inexpensive strategies to reduce the risk of HCAP in patients with severe tetanus would be valuable.

Positioning of mechanically ventilated patients in the semi-recumbent position at 30–45° is now generally recommended as a pneumonia preventative measure.^8^, ^9^, ^10^ In an unpublished pilot study conducted by our group in 20 patients with severe tetanus at the Hospital for Tropical Diseases (HTD) in Ho Chi Minh City, Vietnam, patients were unable to tolerate a semi-recumbent position at a 45° angle because of muscle rigidity. However, a 30° angle was tolerated by the patients and did not appear to cause any adverse events such as hypotension. We investigated the hypothesis that the incidence of HCAP in patients with severe tetanus could be reduced by nursing patients in a semi-recumbent position at 30° rather than in the supine position, as was the current ward practice.

## 2 Methods

### 2.1 Study population

The study was conducted at the HTD, Ho Chi Minh City, Vietnam. This 500-bed infectious disease hospital serves the local community and is a specialist referral centre for the surrounding provinces for severe infectious diseases such as tetanus. The hospital admitted 250–300 cases of tetanus each year to a ward exclusively devoted to the management of patients with tetanus. The ward contained a 14-bed intensive care unit (ICU) for adults, children and neonates with severe disease and a separate area for patients with non-severe disease and those in the recovery phase.

Consecutive adults and children (aged ≥1 year) admitted to the ICU with a clinical diagnosis of severe tetanus were eligible. Patients were excluded if they had been in another hospital for more than 24 h prior to admission to HTD, if they had a clinical diagnosis of pneumonia (defined below) at the time of admission, shock refractory to vasoactive drugs or volume therapy, recent ICU stay (<30 days), recent abdominal surgery (<7 days) or were aged under 1 year. For each eligible patient, an opaque envelope containing the next study number was opened containing a random allocation in a 1:1 ratio to either semi-recumbent (30°) or supine (0°) body position. The randomisation was by a computer-generated list by a staff member not otherwise involved in the study. The attending physicians were responsible for enrolling the participants, and recording the clinical data in the individual study notes. Healthcare personnel were instructed not to change the position of the patient, unless for medical requirements. The correctness of the position was checked twice daily by a member of the study team. Semi-recumbent patients were laid supine if the patient had a cardiac arrest, or hypotension developed for longer than 30 min. All patients were supine during tracheostomy and for 30 min afterwards.

### 2.2 Patient management

Wounds, if present, were cleaned and debrided, equine tetanus antitoxin was administered in a dose ranging from 500 to 100 IU/kg depending on the extent of the disease and penicillin or metronidazole was given (penicillin 100 000–200 000 IU/kg/day or metronidazole 1600 mg/day rectally) for 7–10 days, changing to an oral preparation when the patient was well enough. Benzodiazepines (diazepam or midazolam 20–240 mg/day either as a bolus or by i.v. infusion) were given to control muscle spasm and hypertonia. The indications for a surgical cuffed tracheostomy were acute airway obstruction due to laryngeal spasm, frequent spasms interfering with respiration or to facilitate mechanical ventilation. No patients were orally intubated and no form of subglottic suction or selective digestive tract decontamination was used. Arterial blood gases and peripheral oxygen saturations were monitored regularly. In severe tetanus, the non-depolarizing neuromuscular blocking agent pipecuronium was used, using bolus doses titrated against spasm. Autonomic instability was treated with increased sedation, morphine (20–60 mg/day intramuscularly), calcium antagonists, digoxin, volume expansion or inotropes (norepinephrine or dopamine) according to the clinical situation. Intermittent enteral nutrition was administered through a large bore nasogastric tube in those patients unable to swallow. An X-ray was used to determine correct placement of the tube before feeding commenced. Patients with a history of previous gastric ulceration continued to receive their regular medication, and those who developed gastrointestinal bleeding during the course of their admission were commenced on stress ulcer prophylaxis with either an H2 antagonist or sucralfate. Standard measures for general critical care and prevention of nosocomial pneumonia were employed and a pressure area care protocol was followed in all patients. Closed suction was used for bronchial toilet. On average there were two patients for each nurse in the ICU.

Admission clinical features, the presence of underlying disease, daily progress, the need for a tracheostomy and mechanical ventilation, duration and type of nasogastric intubation, type of stress ulcer prophylaxis, sedative treatment administered, intercurrent infections antimicrobial treatment given, the cost of antimicrobials given and the duration of ICU and hospital stay were collected prospectively on a dedicated study form. At the time of admission to the ICU, blood was taken for haematocrit, white cell count, platelet count and creatinine and a chest X-ray performed. The tetanus severity score (TSS) was determined for the time of admission with a cut-off point TSS ≥8 as predictive of death.^11^ Older age was defined as aged >60 years; prolonged ventilation was defined as mechanical ventilation >7 days; hypotension was defined in adults as a systolic blood pressure ≤80 mmHg and in children as a systolic blood pressure ≤70 mmHg; the presence of autonomic instability was diagnosed by the attending physician on the basis of the presence of lability in the heart rate, blood pressure, temperature or excessive sweating.

### 2.3 Pneumonia surveillance

Surveillance for pneumonia or other infection was conducted daily until death or 72 h after the patient had left the ICU. Patients with clinically suspected pneumonia were investigated with a chest X-ray, white blood cell count, blood culture and non-bronchoscopic bronchial lavage. Clinical pneumonia was defined by the presence of new and persistent infiltrates on chest X-ray, considered likely to be associated with pulmonary infection, and at least two of the following three criteria: temperature of ≥38 °C, white blood cell count ≤4 × 10^9^ or ≥12 × 10^9^/l or the presence of purulent tracheal secretions. The microbial cause of the pneumonia was determined by the isolation of at least one pathogenic microorganism in a blood culture or at least one pathogenic microorganism in the culture of the non-bronchscopic lavage with the bacterial growth ≥10^5^ colony forming units (CFU)/ml. Community-acquired pneumonia was defined as pneumonia developing within 48 h of admission to any hospital and HCAP as pneumonia developing more than 48 h after admission to any hospital. The diagnosis of pneumonia was confirmed by an independent physician, not otherwise involved in the daily conduct of the study.

### 2.4 Microbiological methods

Blood, 5–8 ml (for adults) or 2–5 ml (for children), was inoculated into BACTEC plus aerobic bottles (Becton Dickinson, Sparks, MD, USA). These bottles contain a resin to adsorb antimicrobials. The bottles were incubated at 37 °C in the BACTEC 9050 automated analyser for 5 days and subcultured when the machine indicated a positive signal.

Patients were pre-oxygenated prior to the non-bronchoscopic bronchial lavage.^12^ They were already sedated by the tetanus therapy. Secretions in the trachea and tracheostomy were removed by sterile suction. A standard 50 cm, 14-gauge tracheal aspiration catheter (Argyle Sherwood Medical, London, UK) was attached to a 20 ml syringe filled with 20 ml of sterile saline (10 ml for children). The distal end was lubricated with sterile gel, introduced via the tracheostomy tube and advanced until significant resistance was encountered. The saline was instilled over 10–15 s, withdrawn 1–2 cm and then immediately re-aspirated and the catheter was removed. Generally 5–10 ml of fluid was recovered. No further aspiration was attempted during removal of the catheter to avoid contamination with tracheal secretions.

Samples were processed in the laboratory within 1 h of collection. A Gram stain and Ziehl–Neelsen stain was prepared from the lavage fluid, which was then mixed with an equal volume of freshly prepared dithiothreitol (Sputasol; Oxoid, Basingstoke, UK). The mixture was left at room temperature for 10 min during which time it was shaken vigorously on three occasions. Three serial tenfold dilutions were made by transferring 1 ml of the mixture to 9 ml of maximum recovery diluent (Oxoid, Basingstoke, UK). An aliquot of 20 μl of the original homogenised sample and each of the three dilutions was inoculated on half a plate each of the following media: 5% sheep blood agar, heated blood agar, a selective pneumococcal agar (incubated at 37 °C in 5% CO_2_ for up to 48 h), a MacConkey agar and a further MacConkey agar containing 4 mg/l of gentamicin (incubated at 37 °C in air for up to 48 h) (all media from Oxoid, Basingstoke, UK). Specific cultures for *Legionella* species and *Mycobacteria* spp. were not performed. After 24 and 48 h incubation colonies on each of the plates were counted and converted to a bacterial concentration in CFU)/ml of original lavage fluid.

Isolated organisms were identified by standard laboratory methods using API identification kits (Bio-Mérieux, Basingstoke, UK) when necessary. The following organisms when isolated in the non-bronchial lavage were considered non-pathogenic: *Streptococcus* spp. except *S. pneumoniae*, coagulase negative staphylococci, *Neisseria* spp. and *Candida* spp. Antimicrobial susceptibility testing was performed by the modified Kirby-Bauer method and interpreted according to CLSI (formerly NCCLS) guidelines.^13^ The antimicrobial therapy of the patients was adjusted in the light of the microbiology results.

### 2.5 Sample size and statistical analysis

The aim of the study was to assess the frequency and rate of development of clinically suspected and microbiologically confirmed HCAP in tetanus patients admitted to the ICU nursed in a semi-recumbent or supine body position. The frequency of clinically and microbiologically confirmed HCAP was defined as the number of cases per 100 patients and the rate as the number of cases per 1000 ICU days and per 1000 ventilated days. Patients at risk of developing HCAP were those who had been in hospital for at least 2 days without developing pneumonia. Analysis of admissions to the ward during 1998 and 1999 had shown that approximately 85% of patients admitted to the ICU were at risk, and 39% developed HCAP. In order to show a 50% reduction in the frequency of HCAP in those patients nursed in a semi-recumbent position 190 at-risk patients (95% confidence level, 80% power) would be required. We planned to conduct an analysis when 230 patients had been recruited to the study. A secondary end-point was a comparison of the mortality in each group and this was performed on an intention-to-treat basis. Patients either died in hospital, or were taken by the relatives to die at home when there was no further treatment possible and no likelihood of survival in the view of the attending physician. Those taken home to die were recorded as deaths.

Categorical variables were compared using the χ^2^ test or Fisher's exact test. Non-parametric data was compared using the Mann-Whitney *U* test. Risk factors for the development of HCAP and death were calculated by univariate and multivariate methods. Analysis was performed using SPSS version 18.0 (SPSS Inc., Chicago, IL, USA) and EpiInfo v6 (CDC, Atlanta, GA, USA).

## 3 Results

### 3.1 Study population

**Figure 1:**
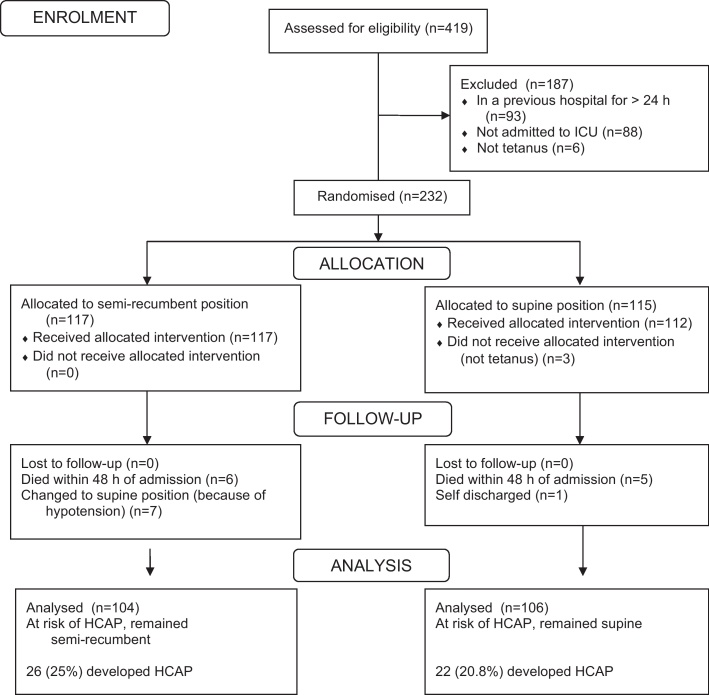
CONSORT flow chart demonstrating recruitment of patients to the study. HCAP: healthcare-associated pneumonia; ICU: intensive care unit.

There were 419 admissions (excluding neonates) to the tetanus ward between August 2000 and March 2002. Six patients were immediately excluded as they did not have tetanus, 88 were not severe enough to require admission to the ICU and 93 had been in a previous hospital for >24 h. A total of 232 patients were entered into the study and randomised (Figure 1): 115 patients were randomised to be nursed in a supine position and 117 to be nursed in a semi-recumbent position. Three supine patients were subsequently considered not to have tetanus and excluded. The only important difference in the characteristics of the two groups of patients, at the time of admission, was that a significantly higher proportion of semi-recumbent patients had previously received an antimicrobial (Table 1). There was no significant difference in the TSS between the two groups.

**Table 1:** Comparison of the admission characteristics of all patients recruited into the study

	Supine (n = 112)	Semi-recumbent (n = 117)	p-value
Mean (range) age (years)	34 (9–67)	38 (13–69)	0.159
Male	87 (77.7%)	96 (82.1%)	0.409
Transferred from another hospital	84 (75.0%)	91 (77.7%)	0.621
Prior antibiotics	20 (17.9%)	37 (31.6%)	0.016
Underlying medical condition	10 (8.9%)	7 (6.0%)	0.395
Injecting drug user	6 (5.4%)	5 (4.3%)	0.701
Smoker	52 (46.4%)	61 (52.1%)	0.388
Vaccination as a child	5 (4.5%)	6 (5.1%)	0.814
Entry site			
Foot	50 (44.6%)	59 (50.4%)	0.381
Unknown	16 (14.3%)	21 (17.9%)	0.452
Injection	6 (5.4%)	4 (3.4%)	0.473
Other	40 (35.7%)	33 (28.2%)	0.223
Mean (range) incubation period (days)	7 (4–13)	7 (4–23)	0.099
Mean (range) period of onset (h)	24 (12–79)	48 (12–96)	0.689
History of spasms	51 (45.5%)	50 (42.7%)	0.670
History of sweating	12 (10.7%)	18 (15.4%)	0.295
Generalised spasms	110 (98.2%)	110 (94.0%)	0.172
Mean (range) tetanus severity score	2.31 (−6 to 25)	1.91 (−5 to 22)	0.582
Mean (range) white cell count (×10^9^/l)	9.5 (5.3–14.7)	8.4 (5.5–14.7)	0.503
Mean (range) creatinine (mg/%)	1.0 (0.7–1.3)	1.0 (0.7–1.4)	0.359

Data are number (%) unless otherwise indicated.

### 3.2 Pneumonia surveillance

A clinical diagnosis of pneumonia was made in 55 patients and a microbiological diagnosis in 45 (Table 2). Of the 55 patients with pneumonia 53 (96%) had a tracheostomy at the time and 50 (91%) were receiving mechanical ventilation. There was no significant difference in the overall number of patients with a clinical or microbiological diagnosis of pneumonia between each group. The frequency of pneumonia in the supine group was lower than we had expected, although the range of organisms isolated was typical of our previous experience on the ward (Table 2).

**Table 2:** Clinical and microbiological pneumonia in 229 study patients

	Supine	Semi-recumbent	Odds ratio (95% CI)	p-value
	(n = 112)	(n = 117)		
Patients with clinical diagnosis of pneumonia	26 (23.2%)	29 (24.8%)	0.92 (0.48–1.76)	0.78
Patients with microbiological diagnosis of pneumonia	19 (17.0%)	26 (22.2%)	0.72 (0.35–1.45)	0.32
Organisms isolated^a^				
*Pseudomonas aeruginosa*	8	11		
*Klebsiella* spp.	10 (1)	11 (1)		
*Acinetobacter* spp.	9 (1)	9		
*Streptococcus pneumoniae*	3	3		
*Staphylococcus aureus*	3	1		
*Haemophilus influenzae*	1	0		
Patients who developed HCAP^2^ (>48 h after admission to hospital)	22/106 (20.8%)	26/104 (25.0%)	0.79 (0.39–1.57)	0.46
Patients with a tracheostomy who developed HCAP	22/49 (44.9%)	26/59 (44.1%)	1.03 (0.45–2.38)	0.93
Ventilated patients who developed HCAP	21/37 (56.8%)	24/44 (54.5%)	1.09 (0.41–2.90)	0.84
HCAP rate/1000 ICU days	13.9	14.6		0.48
HCAP rate/1000 ventilated days	39.2	38.1		0.72
Mean (range) cost of antimicrobials for pneumonia treatment (US$/patient)	228 (56–611)	215 (9–933)		0.84

Data are number (%) unless otherwise indicated.

HCAP: healthcare-associated pneumonia; ICU: intensive care unit.

a Organisms isolated from blood, or from the non-bronchoscopic lavage (≥10^5^ CFU/ml). Number in parentheses refers to the isolation of the organism from blood culture.

Five patients randomised to the supine position died within 48 h of admission and one patient self-discharged on the second day of admission. Six patients randomised to the semi-recumbent position died within 48 h of admission and seven patients had to change position to supine, one because of a cardiac arrest on day 1 and six because they developed hypotension at some point between days 2 and 6. Therefore, 106 supine patients and 104 semi-recumbent patients were eligible for analysis of the frequency and rate of HCAP (Figure 1; Table 2). This was more than the intended sample size of 190 at-risk patients. The proportion of patients with HCAP was 22/106 (20.8%) in the supine group and 26/104 (25.0%) in the semi-recumbent group [odds ratio (OR) 0.79, 95% CI 0.39–1.57, p = 0.46). In the patients treated with a tracheostomy the corresponding proportions were 22/49 (44.9%) vs 26/59 (44.1%) (OR 1.03, 95% CI 0.45–2.38, p = 0.93) and for the patients requiring mechanical ventilation the proportions were 21/37 (56.8%) vs 24/44 (54.5%) (OR 1.09, 95% CI 0.41–2.90, p = 0.84). There were also no significant differences in the rates of HCAP/100 ICU days and HCAP/1000 ventilated days. HCAP only developed in the patients managed with a tracheostomy. In this group of patients, by multivariate analysis the development of clinical pneumonia was independently associated with older age (p = 0.086) and duration of mechanical ventilation for more than 7 days (p < 0.001).

### 3.3 Outcome and adverse events

The proportion of patients who required a tracheostomy, and the overall frequency of complications, was significantly greater by univariate analysis in the patients nursed in the semi-recumbent position compared with those nursed in the supine position (Table 3). The mortality in the patients randomised to the supine position was 11/112 (9.8%) compared with 17/117 (14.5%) in those randomised to the semi-recumbent position (OR 0.64, 95% CI 0.27–1.53, p = 0.277). Other outcome variables were similar in each group. Independent risk factors associated with a fatal outcome by multivariate analysis were an older age (p < 0.001), current or previous injecting drug abuse (p < 0.001) and the occurrence of autonomic instability (p < 0.001). In the 36 patients with a TSS ≥8, the mortality was 19 (52.8%) compared with 9 (4.7%) in the 193 patients with a TSS < 8 (OR 22.9, 95% CI 8.2–65.4, p < 0.001).

**Table 3:** Outcome in 229 study patients

	Supine	Semi-recumbent	Odds ratio (95% CI)	p-value
	(n = 112)	(n = 117)		
Any complication	57 (50.9%)	76 (65.0%)	0.56 (0.32–0.98)	0.031
Tracheostomy used	51 (45.5%)	69 (58.9%)	0.58 (0.33–1.02)	0.042
Mechanical ventilation	39 (34.8%)	53 (45.2%)	0.65 (0.37–1.14)	0.106
Gastrointestinal bleed	30 (26.8%)	34 (29.1%)	0.89 (0.48–1.66)	0.701
Pneumonia	26 (23.2%)	29 (24.8%)	0.92 (0.48–1.76)	0.781
Autonomic instability	14 (12.5%)	17 (14.5%)	0.84 (0.37–1.91)	0.654
Episode of hypotension	11 (9.8%)	20 (17.1%)	0.53 (0.22–1.23)	0.108
Urinary tract infection	12 (10.7%)	7 (6.0%)	1.89 (0.66–5.55)	0.194
Bacteraemia	8 (7.1%)	6 (5.1%)	1.21 (0.38–3.86)	0.525
Wound infection	5 (4.5%)	11 (9.4%)	0.45 (0.12–1.47)	0.143
Pressure sore	5 (4.5%)	7 (6.0%)	0.73 (0.18–2.79)	0.606
Renal failure	1 (0.9%)	2 (1.7%)	0.52 (0.01–1.11)	1.00
Nasogastric tube used	77 (68.8%)	93 (79.5%)	0.57 (0.30–1.80)	0.063
Mean (range) dose of benzodiazepine used (mg/kg/day)	0.98 (0.50–1.46)	0.89 (0.44–1.47)		0.482
Mean (range) dose of pipecuronium used (mg/kg/day)	0.41 (0.04–1.08)	0.59 (0.20–0.97)		0.818
Stress ulcer prophylaxis given	26 (23.2%)	28 (23.9%)	0.96 (0.50–1.85)	0.898
Mean (range) duration on ICU (days)	15 (2–66)	17 (1–108)		0.197
Mean (range) duration in hospital (days)	27 (3–121)	29 (3–108)		0.468
Mortality	11 (9.8%)	17 (14.5%)	0.64 (0.27–1.53)	0.277

Data are number (%) unless otherwise indicated.

ICU: intensive care unit.

## 4 Discussion

In this study a semi-recumbent (30°) or supine nursing position for patients with severe tetanus had no effect on the frequency and rate of HCAP. This result contrasts with two previous studies in general ICU patients. A multivariate analysis of 277 patients requiring mechanical ventilation found that a supine head position during the first 24 h of mechanical ventilation was independently associated with ventilator-associated pneumonia (VAP) and mortality.^14^ A randomised controlled trial in which ventilated patients on a general ICU were randomised to nursing in a semi-recumbent (45°) versus a supine position reduced the frequency of HCAP from 34% to 8% (p = 0.003) and microbiologically confirmed pneumonia from 23% to 5% (p = 0.018).^15^ This study, which was stopped before the planned sample size had been reached, showed that supine body position, enteral nutrition, mechanical ventilation for 7 days or more and a Glasgow Coma Score of less than 9 were independent risk factors for HCAP. A subsequent randomised trial comparing nursing ventilated patients at a 45° semi-recumbent position versus 10° in the control group failed to prevent the development of VAP.^16^ In that study, in which bed elevation was monitored by a transducer with pendulum, it was observed that it proved impossible to maintain the targeted backrest elevation of 45° for semi-recumbent positioning and the mean achieved treatment position was 28°.

The oropharynx of patients who have a tracheostomy or who are mechanically ventilated, rapidly become colonised with an abnormal bacterial flora, particularly Gram-negative bacteria. Reflux of colonised gastric contents into the oropharynx probably contributes to this process. Subsequent aspiration of these organisms into the respiratory tract is suggested to be part of the pathogenic process leading to HCAP. Studies with radioactively labelled gastric contents indicate that positioning ventilated patients in a semi-recumbent position reduces reflux into the oropharynx and subsequent aspiration into the lung.^17^, ^18^ This is the rationale for nursing patients in the semi-recumbent position. It is possible that the pathogenesis of pneumonia in tetanus patients may differ from other ventilated patients. Of note all patients in this study who developed HCAP had a tracheostomy, whereas in the other studies the patients were intubated via the oral route.^14^, ^15^, ^16^ Reflux of gastric contents into the oropharynx and subsequent aspiration into the lung may be a less important route by which pneumonia develops on patients with a tracheostomy and exogenous infection via the tracheostomy may be more important than endogenous infection from the oropharynx.^19^ Of note, patients in this setting had a surgical tracheostomy rather than the percutaneous (PERC) tracheostomies more commonly used in ICUs in developed countries.

The 30° angle may be insufficient to prevent the reflux of gastric contents into the oropharynx and subsequent aspiration into the lung. In the study of Drakulovic^15^ the patients were semi-recumbent at 45° whereas in the study of van Nieuwenhoven^16^ it proved impossible to maintain the planned angle 45°, an average treatment position of 28° was the result on day 1 and was down to 23° by day 7.^15^, ^16^ In the current study we aimed for a 30° angle and this was checked twice daily. It was noted that patients tended to slip down the bed and that it was difficult to maintain the 30° elevation. A limitation of this study is that we did not formally document the adherence to the intended degree of elevation. It has also been suggested that maintaining a supine position in the control group as in the study of Drakulovic^15^ led to a higher than normal rate of HCAP than is the case if a smaller 10° angle is maintained as in the study of van Nieuwenhoven.^16^

The rate of HCAP in this study was 38–39/1000 ventilated days. This rate is high compared with developed country settings but within the range reported in mechanically ventilated patients in developing countries.^20^, ^21^, ^22^ It was lower than we had expected based on previous ward experience. In the period leading up to the study several changes were made in the ward infrastructure and nursing care to improve infection control. This may have contributed to the lower pneumonia frequency during the course of the study. The study size as a result, lacked adequate power to show the 50% reduction in pneumonia frequency that was the target. However, at the time of this analysis, there was no suggestion of a lower pneumonia frequency in the semi-recumbent patients.

The development of pneumonia was independently associated with an older age and a longer duration of mechanical ventilation consistent with other studies of pneumonia in patients receiving mechanical ventilation.^14^ We used a blind non-directed bronchial lavage method with quantitative cultures to determine the organism causing pneumonia.^12^ This method was appropriate for the local situation and gave a range of organisms consistent with studies of VAP from other similar locations.^21^, ^22^, ^23^

Mortality in this study was independently associated with older age, current or previous injecting drug misuse and the presence of autonomic instability. Older age is a well recognized risk factor for mortality in the seriously ill and the presence of autonomic instability a risk factor for mortality in patients with severe tetanus.^2^, ^4^, ^5^, ^24^ The association with injecting drug users is likely to be related to the increased mortality in tetanus associated with intramuscular injections.^25^ In this group of patients, the TTS provided a good predictor of mortality. The mortality rate was slightly higher in the patients managed in a semi-recumbent position but this was not an independent risk factor for mortality in multivariate analysis although the study was not powered to look at this outcome. The overall complication rate, and the need for a tracheostomy, was significantly greater in the semi-recumbent patients compared with those in the supine position despite similar admission characteristics. The need for mechanical ventilation, hypotension and autonomic instability also occurred more frequently in the semi-recumbent group but the differences were not significant.

## 5 Conclusion

In summary, this study suggests that nursing patients with severe tetanus in a semi-recumbent position at an elevation of 30° does not prevent the development of HCAP. This result is likely to be generalisable to severe tetanus patients managed in other similar locations but not necessarily to tetanus patients managed in a developed country ICU or to general ICU patients. Alternative strategies are needed to prevent pneumonia in patients with severe tetanus.

## Authors’ contributions

HTL, JP, NTNN, LMY, JJF and CMP conceived the study and wrote the protocol; all authors participated in the conduct of the study; NTNN, LMY, NTB, TTDT, NMD, JIC, LT and CMP contributed to data interpretation and analysis; CMP wrote the first draft of the paper. All authors read and revised the manuscript and approved the final version. CMP and JJF are guarantors of the paper.

## Funding

The study was funded by the Wellcome Trust of Great Britain (grant reference 089276/Z/09/Z). The study sponsors had no role in the study design, the collection, analysis, or interpretation of the data, the writing of the report, or the decision to submit the paper for publication.

## Competing interests

None declared.

## Ethical clearance

The Scientific and Ethical Committee of the Hospital for Tropical Diseases (Ho Chi Minh City, Vietnam) approved the study. Informed verbal consent was obtained before entry into the study from the patient or their relatives if the patient could not provide consent. The study was conducted in compliance with the ICH and Declaration of Helsinki Guidelines and was registered on a clinical trials database (ClinicalTrials.gov Identifier: NCT01331252).
